# GM-CSF perturbs cell identity in mouse pre-implantation embryos

**DOI:** 10.1371/journal.pone.0263793

**Published:** 2022-02-10

**Authors:** Tim Pock, Katharina Schulte, Stefan Schlatt, Michele Boiani, Verena Nordhoff

**Affiliations:** 1 Centre of Reproductive Medicine and Andrology (CeRA), University of Münster, Münster, Germany; 2 Central Animal Facility of the Faculty of Medicine, University of Münster, Münster, Germany; 3 Max Planck Institute for Molecular Biomedicine, Münster, Germany; University of Florida, UNITED STATES

## Abstract

Growth factors became attractive candidates for medium supplementation to further improve the quality of embryo culture and to mimic *in vivo* nutrition. Granulocyte macrophage colony-stimulating factor (GM-CSF) is a cytokine influencing the maternal-fetal interface and supporting placental development in mouse and human. It is expressed in epithelial cells of the endometrium under the regulation of estrogens. The factor is already in clinical use and a large clinical trial showed that, if supplemented to an embryo culture medium, it leads to increased survival of embryos, especially in women with previous miscarriages. Animal and cell culture studies on isolated trophectoderm cells support an effect mainly on cellular expansion. Aim of this study was to investigate, if the supplementation of GM-CSF either in a human ART medium or in a mouse optimized medium, leads to a change in cell number and cell lineages in the early pre-implantation mouse embryo. Our data shows that mouse GM-CSF increased total cell numbers with increasing concentrations. This increase of cell number has not been found in embryos cultured in ART media with or without human GM-CSF (hGM-CSF) or in a mouse medium supplemented with different concentrations of hGM-CSF. The changes were caused by a marked difference in TE and primitive endoderm cell numbers but not due to a change in epiblast cell numbers. Additionally, results show an ectopic expression of NANOG among trophectoderm cells in both, human ART media (with and without GM-CSF) and at increasing concentrations in the mouse and the human GM-CSF supplemented media. In conclusion, we could show that GM-CSF has an effect on cell identity in mice, which might probably also occur in the human. Therefore, we would like to rare awareness that the use of supplements without proper research could bare risks for the embryo itself and probably also in the post-implantation phase.

## Introduction

Since more than four decades fertilization and early embryo development can also take place artificially outside of the body in a petri dish [1]. This artificial *in vitro* period can range from just a few hours up to several days. So, the *in vitro* condition should be as similar as possible to the natural surrounding and should provide all factors the embryo requires for healthy development. All kind of culture media are supposed to offer this near-natural environment, but still, the content and concentration of nutrients is not and may never be completely similar to the natural environment. First attempts in developing optimized media for *in vitro* culture (IVC) of mammalian embryos were made in the 1950s and 1960s [2, 3]. Today numerous single step or sequential culture media are available for IVC of human pre-implantation stage embryos. The choice of a culture medium may affect birthweight and long term development of ART children, which has been systematically investigated through consecutive studies in the same cohort of ART children [4–6]. Still, researchers and media companies aim at improving embryo culture media, so that they do not only mimic *in vivo* nutrition, but also enhance embryo survival and its possibility to succeed in implantation. For this purpose, growth factors became attractive candidates as media supplements. Growth factors play an important and postulated beneficial role for the culture of embryos, they can improve blastocyst rates and increase cell numbers of the developing embryo [7].

Granulocyte-macrophage colony-stimulating factor (GM-CSF), expressed in epithelial cells of the endometrium under the regulation of estrogens, is one of these growth factors, which seems to have beneficial effects on growth and quality of embryos cultured *in vitro*. GM-CSF and other growth factors, like insulin-like growth factor (IGF) or leukemia inhibitor factor (LIF), are found in the female reproductive tract, where they are involved in ovulation, embryo development, embryo implantation, placental growth and are also important for the maternal-fetal interface [8–10]. A number of studies reported several effects of GM-CSF on cell numbers, either in the trophectoderm (TE) or the inner cell mass (ICM) or in both, depending on the animal model which was used for the study [11, 12]. In mice 2 ng/mL GM-CSF had a positive effect on placental growth [13], while high concentrations of GM-CSF (>5 ng/mL) had a negative impact on blastulation rate [14]. In humans, it was shown that embryos cultured in medium containing GM-CSF (2 ng/mL) had an increased number of viable ICM cells and a reduced rate of apoptosis in the TE and the ICM [15]. Only a few studies exist, which show the influence of GM-CSF on the clinical outcome. These studies have shown that GM-CSF may be able to improve pregnancy rates in human ART, especially in women which experienced miscarriages before [16, 17]. However, another study indicates that the use of ART media containing GM-CSF may influence blastulation rate of human embryos, rather than influencing the pregnancy outcome [18].

Summarizing all reports one could infer that the positive influence of GM-CSF is based on an increase of cell numbers, especially in TE, possibly due to less apoptosis. The increase of TE cells may lead to a higher surface area of the outer blastocyst and concomitantly a higher surface area at the implantation site; this might create a higher probability of implantation and leads to fewer miscarriages and more births. But whether GM-CSF has, apart of the increase of cell numbers also other effects, e.g. on cell identity, is not yet known.

Normally, the first cell lineage decision takes place at the morula stage in mice on day 2.5 to 3.0. The blastomeres undergo the process of compaction and either become the cells which form the oligopotent trophectoderm, from which the extra-embryonic tissues or annexes arise, or they form the pluripotent inner cell mass cells resulting in the embryonic lineage, from which the later fetus arises [19, 20]. The differences between ICM and TE are based on distinct gene expression patterns with different sets of genes. In particular, *Oct4* is the best-known marker of the ICM, while *Cdx2* is the adequate marker for TE [21]. A second cell lineage decision occurs directly before implantation at E4.5, separating the ICM into the primitive ectoderm (marker *Sox17*), which will form the later amnion, and the epiblast (marker *Nano*g), which forms the proper embryo [20, 22]. It is assumed that this early cell lineage decision can be influenced *in vitro* by extrinsic factors, like culture media or media supplements, respectively. Thus, it has already been shown that human ART culture media influence early mouse embryo development and prepare embryos differently for post-implantation development [23], whereby, if the embryo can implant successfully, these mouse fetuses develop normally without any constraints [24]. As the impact of GM-CSF on TE and ICM segregation and later ICM lineage segregation into primitive endoderm and epiblast is not yet clear, we hypothesized that GM-CSF could be a modulator of cell lineage decision, and designed this study to test our hypothesis in the mouse as a model for human reproduction.

## Material & methods

### Animals

All mice were kept in the institutional breeding facility and housed in individually ventilated cages under a 12/12 hours light/dark cycle with food pellets (Altromin, Lage, Germany) and water *ad libitum*. Housing and exercise conditions were identical for all animals. C3H male mice and C57Bl/6 mice were mated to generate B6C3F1/N hybrid mice. Female offspring of these hybrids were used as a source of *in-vivo*-fertilized zygotes after mating to C57Bl/6 males. Therefore, the female offsprings were sacrificed by cervical-dislocation. Experimental procedures were performed in compliance with the German Federal Law on the Care and Use of Laboratory Animals (LANUV NRW) and according to Federation of European Laboratory Animal Science Associations (FELASA) recommendations; the animal license was prospectively acquired (LANUV number: 8.87–51.05.20.10.070).

### Zygote collection and embryo culture

Collection of zygotes, embryo culture, and immunohistological analysis were conducted with minor modifications as previously described [23, 25, 26]. Six to ten-week-old B6C3F1 female mice were superovulated by intraperitoneal injections of 5 IU PMSG, followed by a single injection of 10 IU hCG 48 hours later. Females were mated to C57BL/6J males directly after hCG injection. 17 to 18 hours after hCG injection female mice were sacrificed by cervical dislocation and zygotes were collected. At first, mouse zygotes were cultured *in vitro* up to the blastocyst stage (day 4.5) in KSOM(aa) medium without and with increasing concentrations of mGM-CSF (1, 2, 5, 10 and 20 ng/ml; R&D Systems). For further analysis embryos were also cultured in ART culture media, either supplemented with 2ng/mL GM-CSF, which is EmbryoGen®/BlastGen^TM^ (Origio; Group ART+), or without GM-CSF, which is Cleave^TM^/Blast^TM^ (Origio; Group ART-) and in KSOM(aa), supplemented with increasing concentrations of human recombinant GM-CSF (1, 2, 5, 10 and 20 ng/ml; R&D Systems). Zygotes were pooled after collection and randomly distributed to the different protocols: 30 to 50 zygotes were transferred into each well of a 4-well plate, containing 500 μL medium and then incubated at 37°C and 5.5% CO_2_. Embryos were cultured for 96 hours (blastocysts stage at day 4.5) with a medium refreshment step at day 2.5 (S1 Table). After culture in different conditions embryos were collected and subjected to immunohistochemical staining.

### Immunohistochemical staining & analysis

At day 4.5 blastocysts were fixed in 1.5% paraformaldehyde in PBS following permeabilization in 0.1% Triton X-100 in 1x PBS each for 15 minutes at room temperature (RT). Embryos were then put in blocking buffer to block unspecific binding sites (0.1%Tween in 1xPBS, 2% BSA, 2% glycine, 5% donkey serum) for 2h at 4°C. After blocking the embryos were transferred to Tyrode’s acidic solution to remove the *zona pellucida* for 20 to 30 seconds at RT. Blastocysts were transferred to fresh blocking buffer and incubated up to a maximum of 3 days until the antibody staining was performed. Embryos were stained with primary antibodies (anti-CDX2 mouse IgG, abcam; anti-SOX17 goat IgG, R&D Systems; anti-Nanog rabbit IgG, Cosmo Bio Co.) in antibody solution (0.5% BSA, 0.5% glycine, 1.25% donkey serum, 0.1% Tween-20 in 1x PBS). Blastocysts were analyzed using fluorescence microscopy to identify cell lineages. Markers of the three cell lineages trophectoderm (CDX2), primitive endoderm (SOX17) and epiblast (NANOG) enabled selective scoring of absolute cell numbers using Fiji [27].

### Statistical analysis

Absolute numbers of the different cell lineages were analyzed by one-way ANOVA followed by Bonferroni post-hoc test. Significant differences are marked with * for P<0.05, ** for P<0.01 and *** for P<0.001. Data were either presented as box plots showing median and quartiles and the whiskers extend for 1.5x the interquartile distance or only mean values were plotted for direct comparison of different experimental groups.

## Results

**Fig 1:**
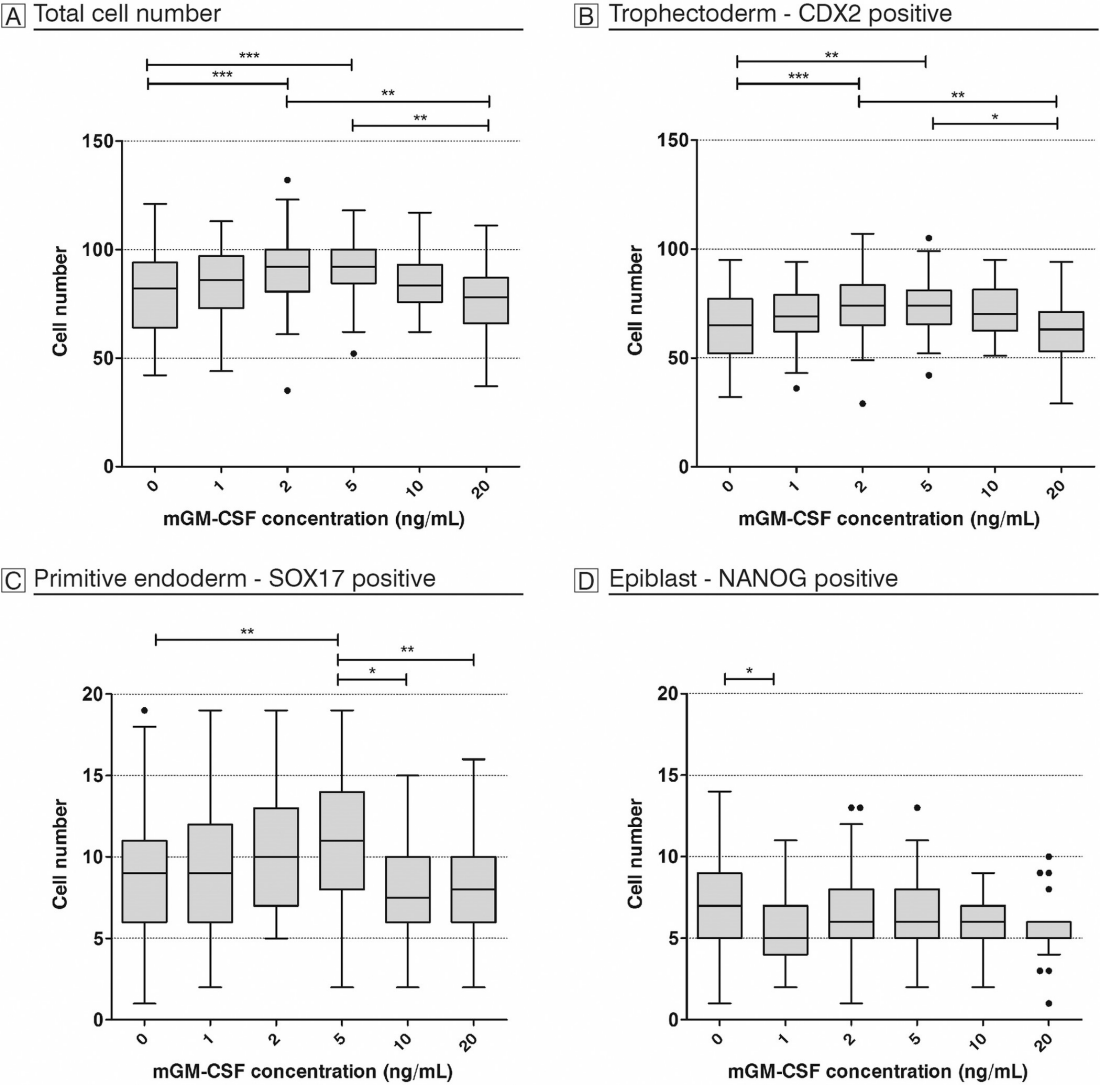
Effect of mGM-CSF: Comparison of absolute cell numbers.

**Fig 2:**
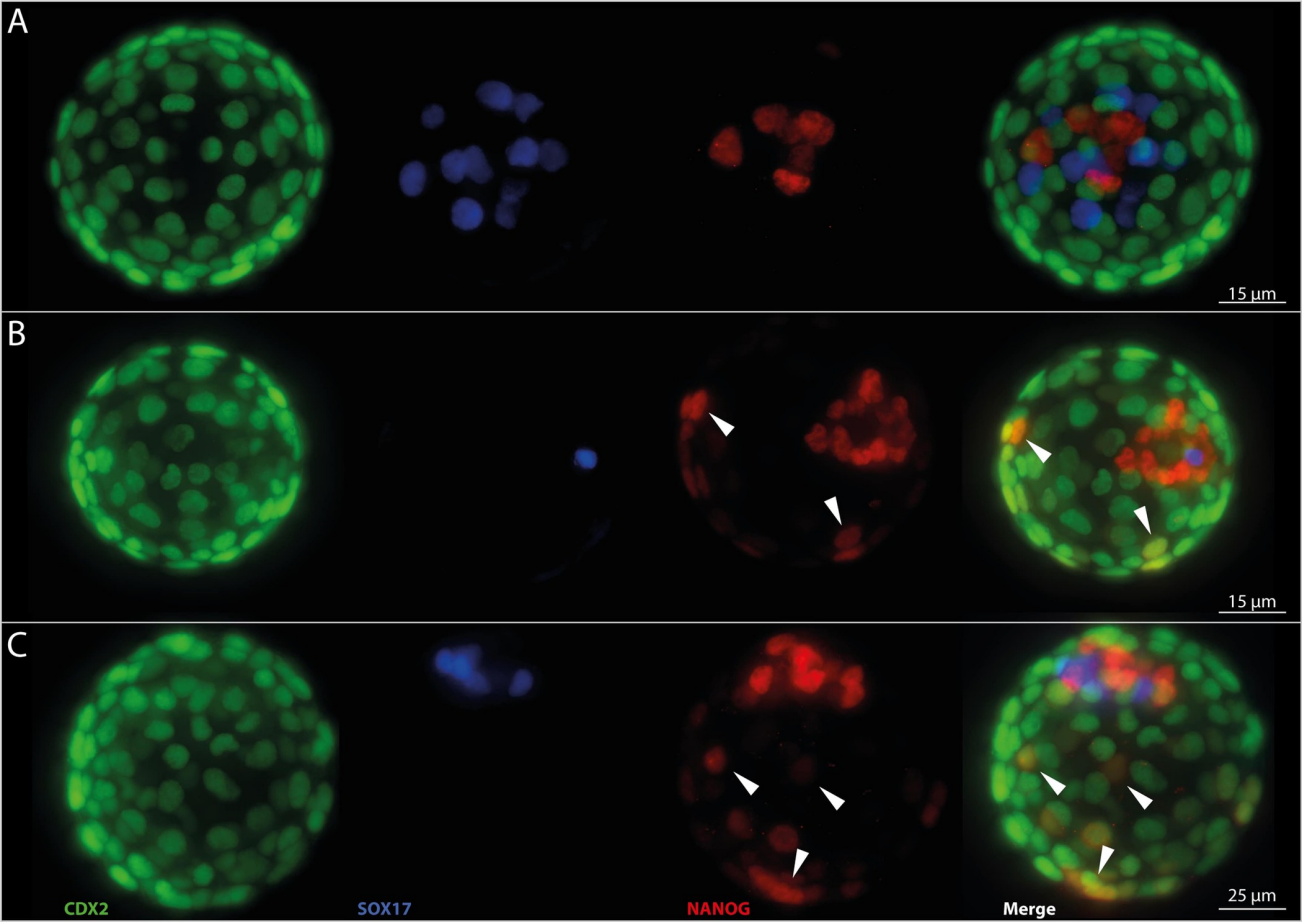
Representative picture of a blastocyst (E4.5) with ectopic expression of NANOG.

**Fig 3:**
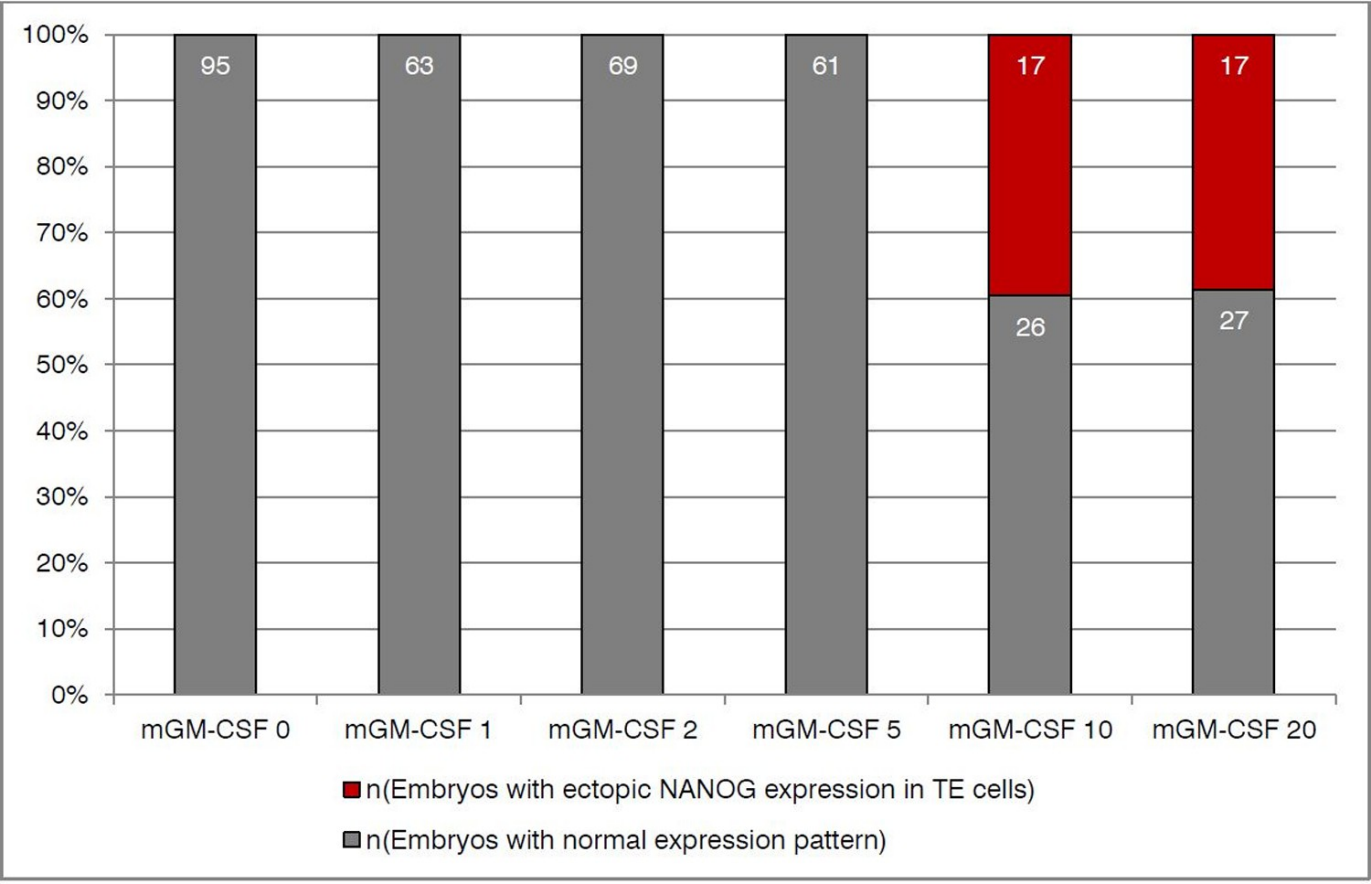
Percentage of occurrence of blastocysts cultured in medium containing mGM-CSF exhibiting a double staining of the cell lineage markers CDX2 and NANOG in trophectoderm cells.

**Fig 4:**
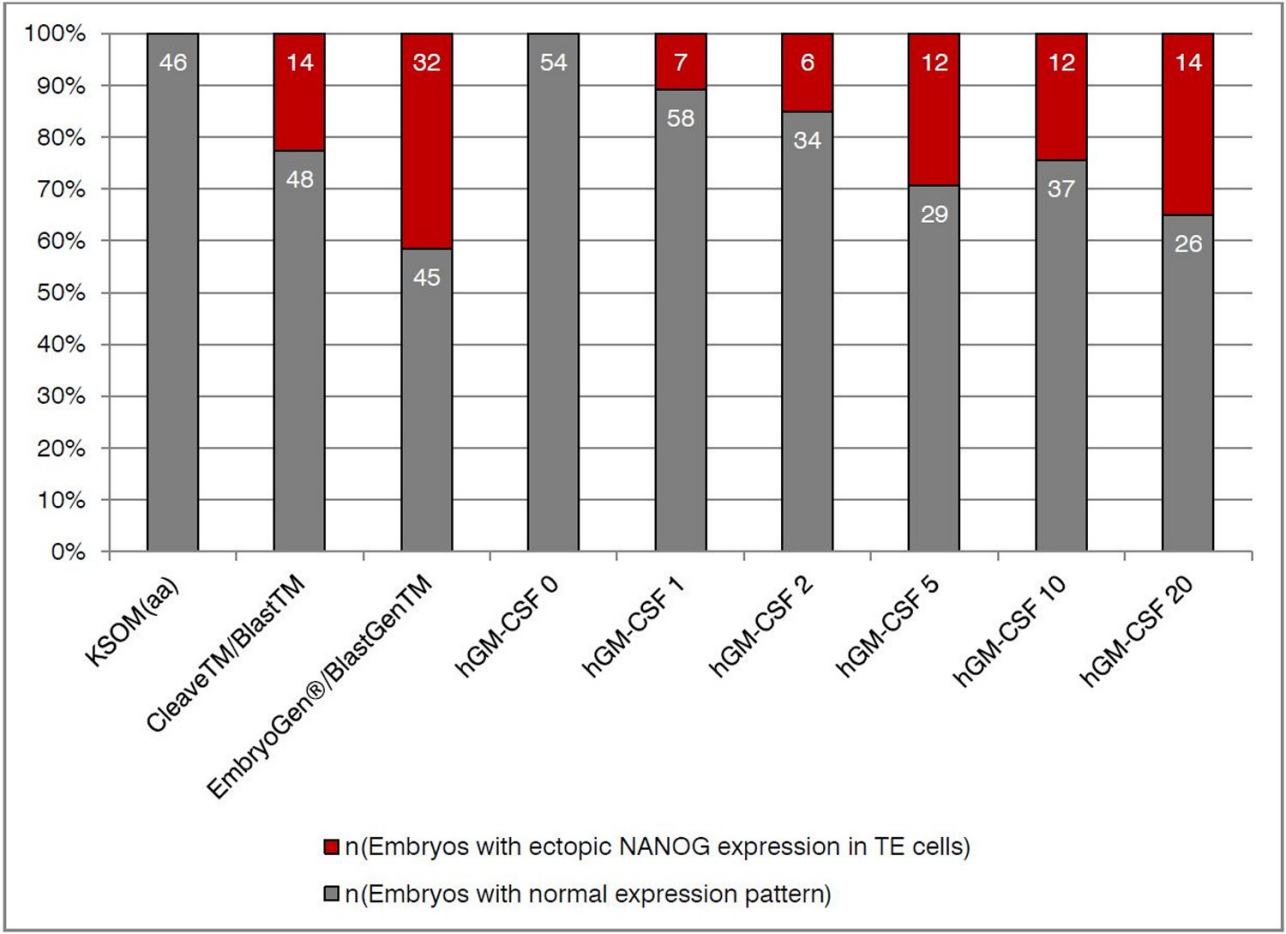
Percentage of occurrence of blastocysts cultured in ART media and medium containing hGM-CSF exhibiting a double staining of the cell lineage markers CDX2 and NANOG in trophectoderm cells.

To evaluate if GM-CSF has an effect on mouse pre-implantation development, we firstly supplemented the mouse optimized medium KSOM(aa) with increasing concentrations of mouse GM-CSF. Thus, we analyzed a total of 375 blastocysts and evaluated total cell count, as well as the number of cells positive for CDX2, SOX17, and NANOG (Fig 1). We found changes in cell numbers as a result of GM-CSF culture. Total cell count and number of TE (CDX2-positive) cells both increased significantly between control and 2 and 5ng/mL. However, cell numbers increased up to 2 ng/ml in comparison to control and went down again with a further increase of mGM-CSF concentration beyond 5ng/mL. In the inner cell mass, the number of SOX17-positive cells increased also up to a concentration of 5 ng/mL mGM-CSF compared to control and decreased again substantially with increasing GM-CSF concentration, while the number of epiblast cells (NANOG) remained equal in all concentrations. Interestingly, next to the normal CDX2 pattern located in TE cells, we detected an ectopic expression of NANOG (superimposed on CDX2) in a non-negligible proportion of embryos that were cultured in KSOM(aa) with mGM-CSF. Overall we found 34 embryos (9%) with an ectopic expression of NANOG among CDX2 positive trophectoderm cells, with maximal occurrence of 40% in the highest concentrations of mGM-CSF (KSOM(aa) +10 ng/mL: 40%; +20 ng/mL: 39%; Figs 2 and 3). This finding was unexpected and we wanted to see if this atypical expression pattern of pluripotency and trophectodermal marker could also be detected in a commercially available human ART culture medium supplemented with hGM-CSF. Therefore, we incubated mouse embryos in a human ART medium with hGM-CSF and as control either in the same medium with equal formula but without growth factor, or in the KSOM(aa). And again, we found a total of 46 embryos with ectopic expression (25%) among 185 analyzed ART blastocysts. We had a high frequency of co-expression (14%) already in the normal human culture medium (CleaveTM/BlastTM; Group ART-); this effect became even more obvious when embryos were cultured in the presence of hGM-CSF (32%, EmbryoGen®/BlastGenTM; Group ART+). Both findings were in clear contrast to the complete lack of co-expression in the optimized mouse medium (KSOM(aa)). This was unexpected since GM-CSF is considered to protect blastocysts from adverse effects of cell stress [28] and KSOM(aa) is a less refined and thereby ’more stressful’ medium compared to the ART+ group and the ART- group. To clarify if this effect was also dose-dependent, we used an interspecies approach and incubated mouse embryos in KSOM(aa) medium supplemented with increasing concentrations of hGM-CSF as done before with mGM-CSF. And again, in 298 analyzed embryos cultured in KSOM(aa) with human GM-CSF we found a total of 51 embryos with ectopic NANOG expression (18%). Increasing concentrations of hGM-CSF also increased the number of embryos with ectopic expression. Opposite to mGM-CSF cultivation, embryos cultured with human GM-CSF showed abnormalities already from the lowest concentration onwards (0 ng/mL: 0%; 1 ng/mL: 11%; 2 ng/mL: 15%; 5 ng/mL: 29%; 10 ng/mL: 24%; 20 ng/mL: 35%; Fig 4). Thus, the effect was more pronounced in the heterospecific experiment compared to the iso-specific experiment between the source of embryos (mouse) and source of factor (mouse or human). Interestingly, total cell numbers and cell lineage numbers did not differ among different concentrations of human GM-CSF or between ART media with and without growth factor (S1 and S2 Figs, S2 and S3 Tables). Altogether, this shows that GM-CSF had not only an effect on cell lineage numbers but also on cell identity. Our data hypothesize that factors e.g. the growth factor GM-CSF may modulate pre-implantation development, possibly creating also unwanted effects.

Effect of mGM-CSF: comparison of absolute numbers of A) total cells B) CDX2 positive trophectoderm cells, C) SOX17 positive primitive endoderm cells and D) NANOG positive epiblast cells in five different mGM-CSF concentrations (1, 2, 5, 10 and 20 ng/mL) and the *in vitro* control medium, 0 ng/mL (KSOM(aa)). Boxes illustrate median and quartiles and the whiskers extend for 1.5x the inter quartile distance; dots not included between the whiskers indicate outliers. Significance values: P<0.05 (*), P<0.001 (**) and P<0.0001 (***). One-Way ANOVA followed by Bonferroni post-hoc test. Number of embryos: 0 ng/mL n = 95; 1 ng/mL n = 63; 2 ng/mL n = 69; 5 ng/mL n = 61; 10 ng/mL n = 26; 20 ng/mL n = 27).

Representative fluorescence picture of 4.5 day old embryos with normal cell lineage expression pattern (A) and embryos with ectopic NANOG expression in TE cells (B, C). White arrows highlight exemplary NANOG positive TE cells.

Percentage of occurrence of embryos exhibiting double staining of the cell lineage markers CDX2 and NANOG in trophectoderm cells in KSOM(aa) and all concentrations of mouse GM-CSF (1, 2, 5, 10 and 20 ng/mL). Grey: embryos without double staining; Red: embryos with CDX2 and NANOG positive trophectoderm cells. Bars indicate the percentage occurrence and numbers inside bars indicate absolute values of embryos.

Percentage of occurrence of embryos exhibiting a double staining of the cell lineage markers CDX2 and NANOG in trophectoderm cells in KSOM(aa) and both ART media (ART-: Cleave^TM^/Blast^TM^: ART medium without GM-CSF (Origio); ART+: EmbryoGen®/BlastGen^TM^: ART medium containing 2 ng/mL GM-CSF (Origio) and all concentrations human GM-CSF (1, 2, 5, 10 and 20 ng/mL). Grey: embryos without double staining; Red: embryos with CDX2 and NANOG positive trophectoderm cells. Bars indicate the percentage occurrence and numbers inside bars indicate absolute values of embryos.

## Discussion

The success of ART is a healthy live birth. To reach this goal we try to increase pregnancy rates by supporting the pre-implantation embryo as good as possible. However, it is not only important to support the embryo at its best, but it is also equally important to do no harm to the embryo. This holds true also for a culture medium, it should have no influence on the normal developmental program of an embryo. However, we found that embryo culture media supplemented with the cytokine GM-CSF do influence preimplantation development by enhancing cell numbers, especially TE cells. Beyond that, GM-CSF was also able to induce an atypical expression pattern of pluripotency and trophectodermal marker in the mouse pre-implantation embryo. In our intraspecies approach we saw that mouse GM-CSF clearly had an influence on total cell numbers, they increased with elevated GM-CSF concentrations in comparison to our control medium. These changes were caused by a marked difference of TE and primitive endoderm cell numbers (CDX2 and SOX17 positive) but no change in epiblast cell numbers (NANOG positive). A higher proliferation rate and the subsequent increase in TE cell numbers have also been found in an independent study where TE cells from mice were treated with GM-CSF in a pure cell culture approach [29]. This was also seen in another mouse study; however, they showed that not only TE cell number, but also ICM cells numbers change [14], though, they did not subdivide the ICM cells into epiblast and primitive endoderm cells. Supplementing culture media with a combination of growth factors, including CSF, confirmed the positive effect on TE and total cell numbers [30]. At variance with these findings, others did not find any changes in different concentrations of mouse GM-CSF in human ART culture media at all [31]. What we cannot answer with our experiments is, if the increase of TE and ICM cell numbers by incubation of pre-implantation embryos with GM-CSF is the sole reason for better survival of the embryo. Further animal studies investigating the implantation and post-implantation processes and fetal development would be necessary. However, a glance at human clinical data shows a significant increase in survival and live birth rates, when preimplantation embryos were cultured in media with GM-CSF, especially in women with previous miscarriages [17]. These results could not be confirmed by another study, though they found a difference in blastulation rate of embryos cultivated in media containing GM-CSF [18]. In our study, we saw no difference in development rates among all experimental groups (data not shown), as also shown in another animal study [30]. There is no effect on ploidy rates, as human embryos cultured in ART media with hGM-CSF have the same ploidy rates as embryos cultured without hGM-CSF [32]. The culture of embryos in GM-CSF affects preimplantation development by reducing apoptosis rates and changing cell numbers in the mouse [14, 31, 33] as well as in the human blastocyst [15]. Interestingly, the presence and concentration of human serum albumin (HSA) in a culture medium may play an important role for the occurrence of a GM-CSF effect. While Karagenc et al. found no effect if HSA was present ([31], 2005), Ziebe et al. saw that there was an effect in the presence of HSA which was even enhanced if HSA concentrations were increased from 2 ng/mL to 5 ng/mL [17]. In all our experiments, we used culture media containing HSA (or BSA) and found a clear effect of GM-CSF in our mouse blastocysts. However, while these studies only distinguished between TE and ICM only, our study expanded the analysis into all three different cell lineages and a wider range of different GM-CSF concentrations. What puzzled us the most was the unexpected finding that among all embryos cultured either in mouse or in human GM-CSF supplemented media, we found embryos showing an atypical expression pattern of the different cell lineages. This atypical finding was an ectopic expression of NANOG among CDX2-positive TE cells in both, human ART media (with and without GM-CSF) and at increasing concentrations in the mouse and the human GM-CSF supplemented media. NANOG is a well-known transcription factor, which fulfills a key role in ICM and epiblast lineage establishment and maintenance [34]. It also represses the trophoblast gene *Cdx2* [35] which of course should be silenced in the ICM. We identified among all mGM-CSF groups a total amount of 9% embryos with an ectopic NANOG expression, and actually among the two highest concentrations an amount of 40% of these specific embryos. Furthermore, we identified a total of 18% embryos with ectopic NANOG expression in media supplemented with hGM-CSF. While embryos cultured in the mouse medium supplemented with mouse GM-CSF showed atypical expression only in the highest and possible supraphysiological concentrations, embryos cultured in human GM-CSF showed this co-expression already from the lowest concentration onwards. What is striking is the fact that also embryos with NANOG/CDX2 co-expression in the human ART media show this alteration already in medium without supplementation of GM-CSF. It seems that this specific human ART medium has already the feasibility to change cell fate. The presence of recombinant human insulin in this human ART medium may be part of the explanation for these findings. Variable effects, like a change in cell number or a decrease of blastocyst rates, were shown in animal models when insulin was present in the culture medium. Interestingly, gene expression analysis also indicated an influence on metabolic processes which may influence cell fate in the early embryo [36].

Although the frequency of embryos with atypical expression pattern was different, the absolute number of atypical cells in each embryo was equal and therefore comparable (**S3 Table**). It is remarkable that although human GM-CSF showed no influence on embryo development regarding cell numbers (S3 Fig) at all, it had a dose depended effect on the occurrence of NANOG positive trophectoderm cells in mouse embryos. These findings are divergent from a previous study, where genes regulating the NANOG pathway were downregulated in bovine trophectoderm cells after these embryos were cultured in a medium containing 10 ng/mL GM-CSF [37]. It may be speculated that GM-CSF affects the cell fate specification processes. CDX2 and NANOG are part of a transcriptional network regulating the development of trophectoderm and inner cell mass, and GM-CSF may interrupt these molecular machinery [38]. Two scenarios explaining this ectopic expression are conceivable: 1) an error of programming of cell identity, or 2) a false positioning of correctly programmed cells. Either way, these errors could influence the pre- and post-implantation processes, possibly also in the human. A caveat of our study is that the interspecies combination with mouse embryos in a medium with the human growth factor is rather non-physiological. In addition, we have to be cautious to translate our findings one-to-one to the human situation. Although mouse and human pre-implantation development appear to be very equal, still, a lot of differences exist, e.g. it is believed that the first and second cell lineage decision in the human is not separated as it is in the mouse, it occurs at the same time. Cells in the human embryo seem to retain plasticity longer than the cells in the mouse [39]. The role of insulin and serum itself, which are part of all compositions, remains an open question and could be investigated independently in future attempts. Furthermore, this study cannot predict the fate of cells with ectopic NANOG/CDX2 expression during post-implantation development. Still, we believe that all these considerations do not question the message that GM-CSF has an effect on cell identity, which might probably also occur in the human. While transcriptional profiles seem unaffected by the use of GM-CSF only, a combination of different growth factors may be able to alter these profiles of embryos and may have later developmental impact [30]. We would like to propose that the addition of e.g. growth factors to human culture media should not be done without enough clinical evidence or preceding basic research. Importantly, further work is required to identify exact effects on implantation and fetal development in animal models like mice.

## Supporting information

S1 Fig Effect of ART media: Comparison of absolute cell numbers. Comparison of cell numbers of all analyzed blastocysts, A) total cells, B) CDX2 positive trophectoderm cells, C) SOX17 positive primitive endoderm cells and D) NANOG positive epiblast cells in three different media settings (KSOM(aa): *in vitro* control; ART-; Cleave^TM^/Blast^TM^: ART medium without GM-CSF (Origio); ART+; EmbryoGen®/BlastGen^TM^: ART medium containing 2ng/mL GM-CSF (Origio)). Boxes illustrate median and quartiles and the whiskers extend for 1.5x the interquartile distance; dots not included between the whiskers indicate outliers; P<0.05 (*) significant. Number of embryos: KSOM(aa) n = 46; ART- n = 48; ART+ n = 45.
(TIF)

S2 Fig Comparison of mean absolute numbers of different cell lineages. Comparison of mean absolute numbers of different cell lineages identified by specific cell lineage markers (CDX2: trophectoderm; SOX17: primitive endoderm; NANOG: epiblast) in ART media (ART-: Cleave^TM^/Blast^TM^: ART medium without GM-CSF; ART+: EmbryoGen®/BlastGen^TM^: ART medium with GM-CSF) and different mouse and human GM-CSF concentrations (1, 2, 5, 10 and 20 ng/mL) and the *in vitro* control medium, 0ng/mL (KSOM(aa)).
(TIF)

S3 Fig Effect of hGM-CSF: Comparison of absolute cell numbers. Effect of hGM-CSF: comparison of A) total cells, B) CDX2 positive trophectoderm cells, C) SOX17 positive primitive endoderm cells and D) NANOG positive epiblast cells in five different hGM-CSF concentrations (1, 2, 5, 10 and 20 ng/mL) and the *in vitro* control medium, 0ng/mL (KSOM(aa)). Boxes illustrate median and quartiles and the whiskers extend for 1.5x the inter quartile distance; dots not included between the whiskers indicate outliers. Numbers of embryos: 0 ng/mL n = 54; 1 ng/mL n = 58; 2 ng/mL n = 34; 5 ng/mL n = 28; 10 ng/mL n = 37; 20 ng/mL n = 26).
(TIF)

S1 Table Medium composition of commercially available ART media and KSOM(aa). Medium composition of the commercially available ART media and the control medium KSOM(aa).
(TIF)

S2 Table Mean values of cell lineages, standard deviation and n-values of all experimental groups. Mean numbers, standard deviation and n-values of all media groups and cell lineages identified by specific cell lineage markers (CDX2: trophectoderm; SOX17: primitive endoderm; NANOG: epiblast). Significance values: a: P<0.0001; b, c, e, f: P<0.001; d, g, h, i: P<0.05; One-Way ANOVA followed by Bonferroni post-hoc test.
(TIF)

S3 Table Mean values of all cell lineages, standard deviation and n-values of blastocysts with ectopic NANOG expression in all experimental groups. Mean numbers, standard deviation and n-values of all media groups of embryos with double staining of NANOG in CDX2 positive cells, identified by specific cell lineage markers (CDX2: trophectoderm; SOX17: primitive endoderm; NANOG: epiblast).
(TIF)
