# Supplementary figures and images for: GM-CSF perturbs cell identity in mouse pre-implantation embryos
Source: PLoS One. 2022 Feb 10;17(2):e0263793. doi: 10.1371/journal.pone.0263793 (PMC8830693; doi:10.1371/journal.pone.0263793)

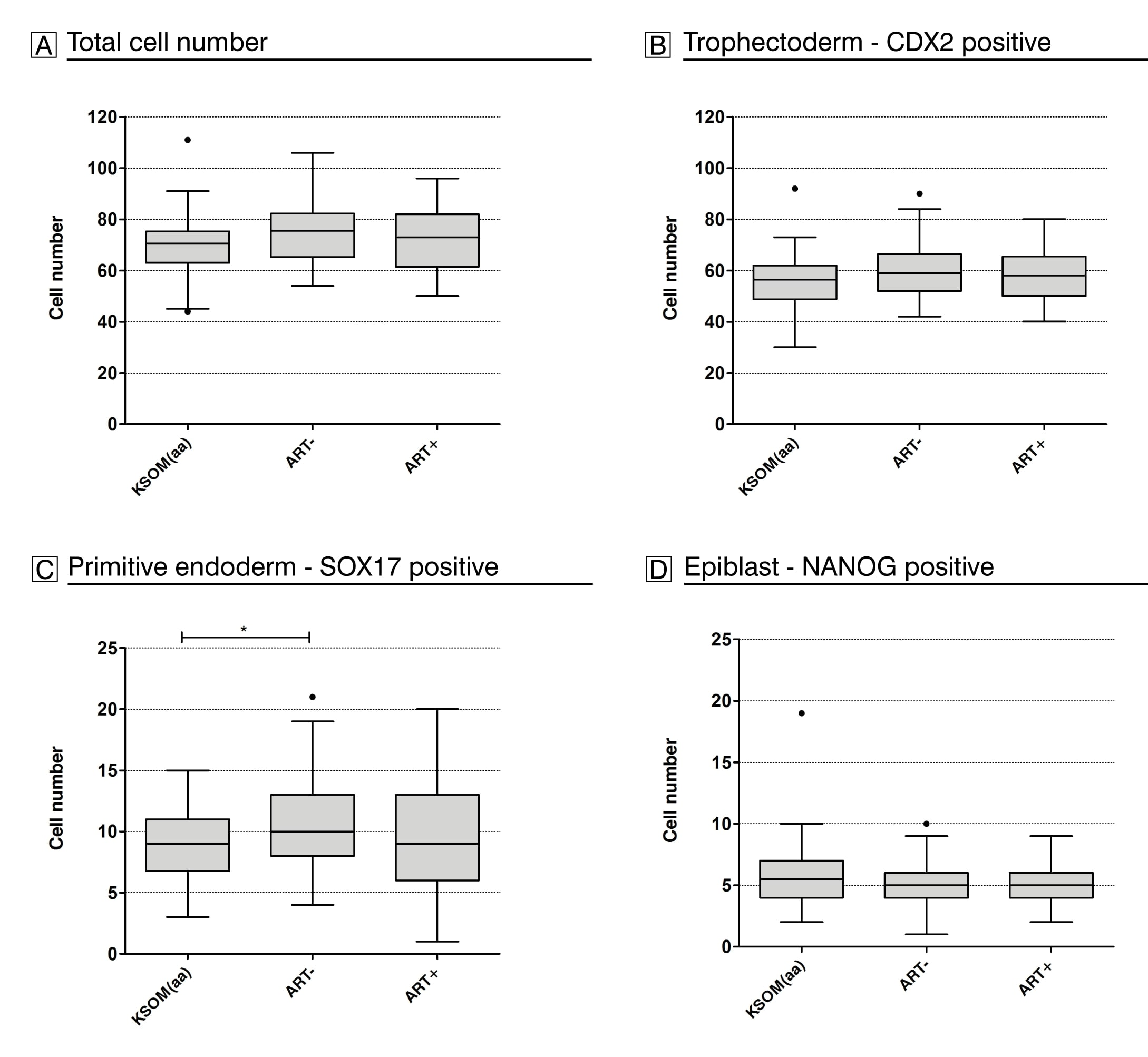

Supplement: S1 Fig — Comparison of cell numbers of all analyzed blastocysts, A) total cells, B) CDX2 positive trophectoderm cells, C) SOX17 positive primitive endoderm cells and D) NANOG positive epiblast cells in three different media settings (KSOM(aa): in vitro control; ART-; CleaveTM/BlastTM: ART medium without GM-CSF (Origio); ART+; EmbryoGen®/BlastGenTM: ART medium containing 2ng/mL GM-CSF (Origio)). Boxes illustrate median and quartiles and the whiskers extend for 1.5x the interquartile distance; dots not included between the whiskers indicate outliers; P<0.05 (*) significant. Number of embryos: KSOM(aa) n = 46; ART- n = 48; ART+ n = 45. (TIF) [file pone.0263793.s001.tif]

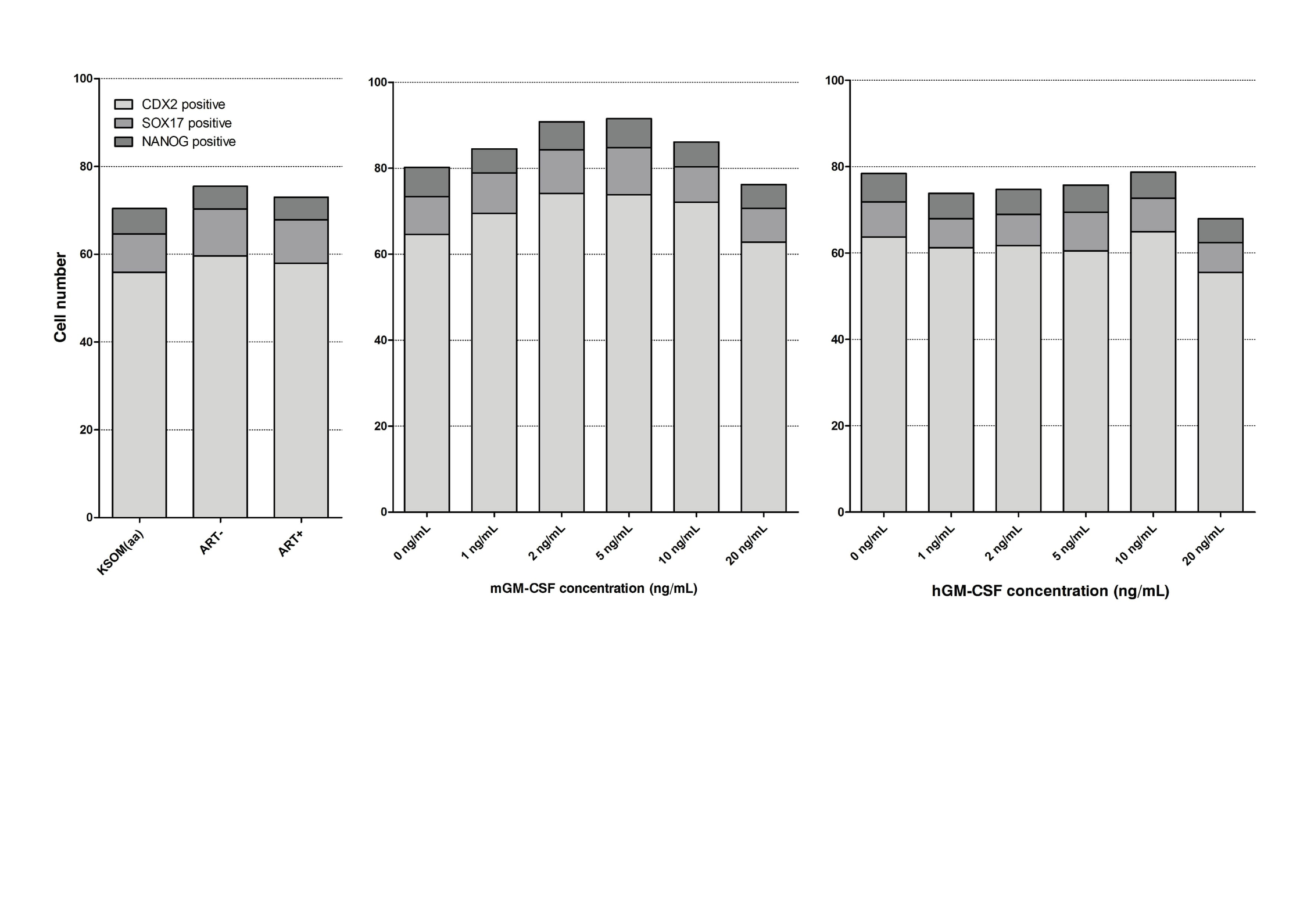

Supplement: S2 Fig — Comparison of mean absolute numbers of different cell lineages identified by specific cell lineage markers (CDX2: trophectoderm; SOX17: primitive endoderm; NANOG: epiblast) in ART media (ART-: CleaveTM/BlastTM: ART medium without GM-CSF; ART+: EmbryoGen®/BlastGenTM: ART medium with GM-CSF) and different mouse and human GM-CSF concentrations (1, 2, 5, 10 and 20 ng/mL) and the in vitro control medium, 0ng/mL (KSOM(aa)). (TIF) [file pone.0263793.s002.tif]

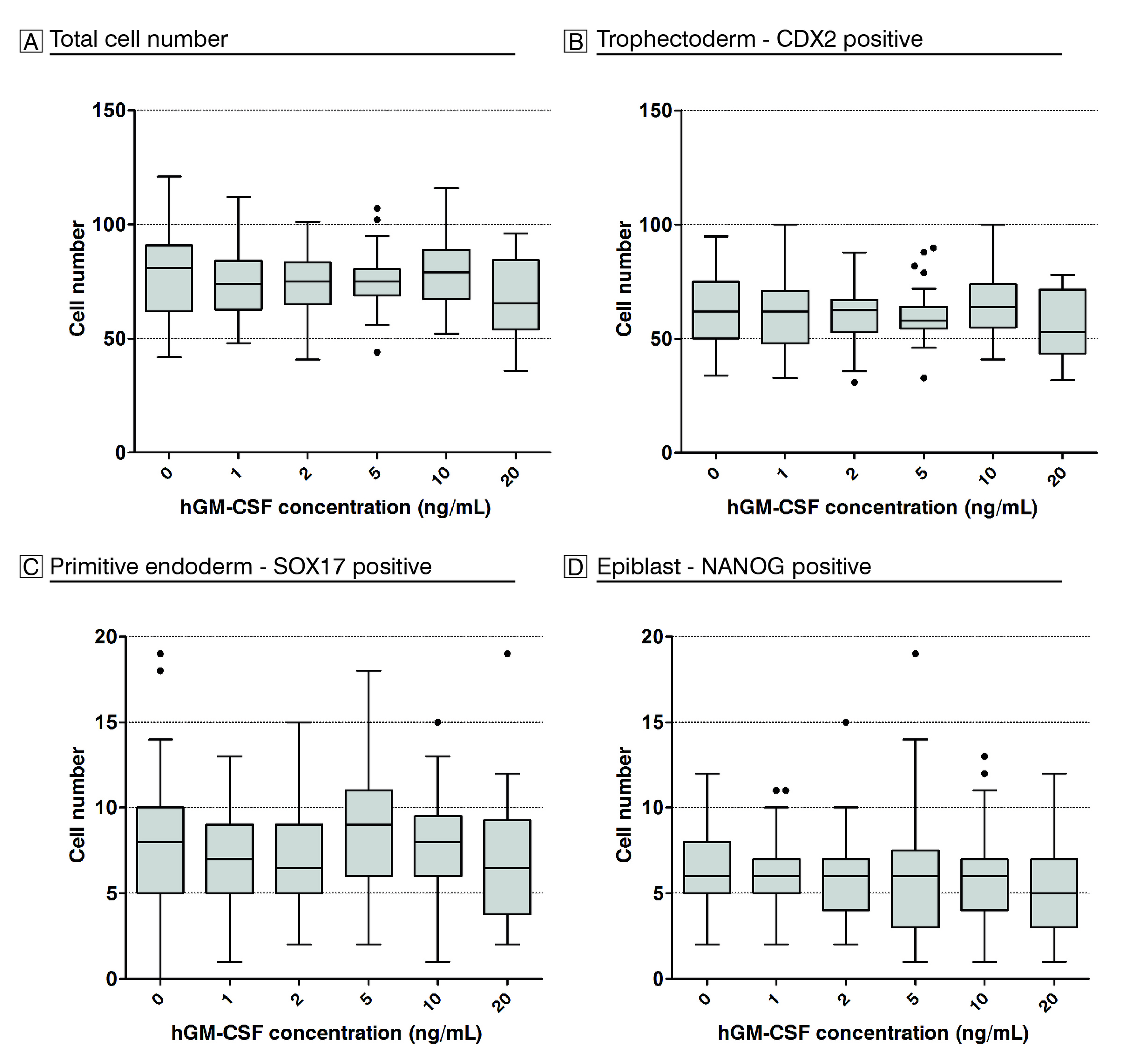

Supplement: S3 Fig — Effect of hGM-CSF: comparison of A) total cells, B) CDX2 positive trophectoderm cells, C) SOX17 positive primitive endoderm cells and D) NANOG positive epiblast cells in five different hGM-CSF concentrations (1, 2, 5, 10 and 20 ng/mL) and the in vitro control medium, 0ng/mL (KSOM(aa)). Boxes illustrate median and quartiles and the whiskers extend for 1.5x the inter quartile distance; dots not included between the whiskers indicate outliers. Numbers of embryos: 0 ng/mL n = 54; 1 ng/mL n = 58; 2 ng/mL n = 34; 5 ng/mL n = 28; 10 ng/mL n = 37; 20 ng/mL n = 26). (TIF) [file pone.0263793.s003.tif]

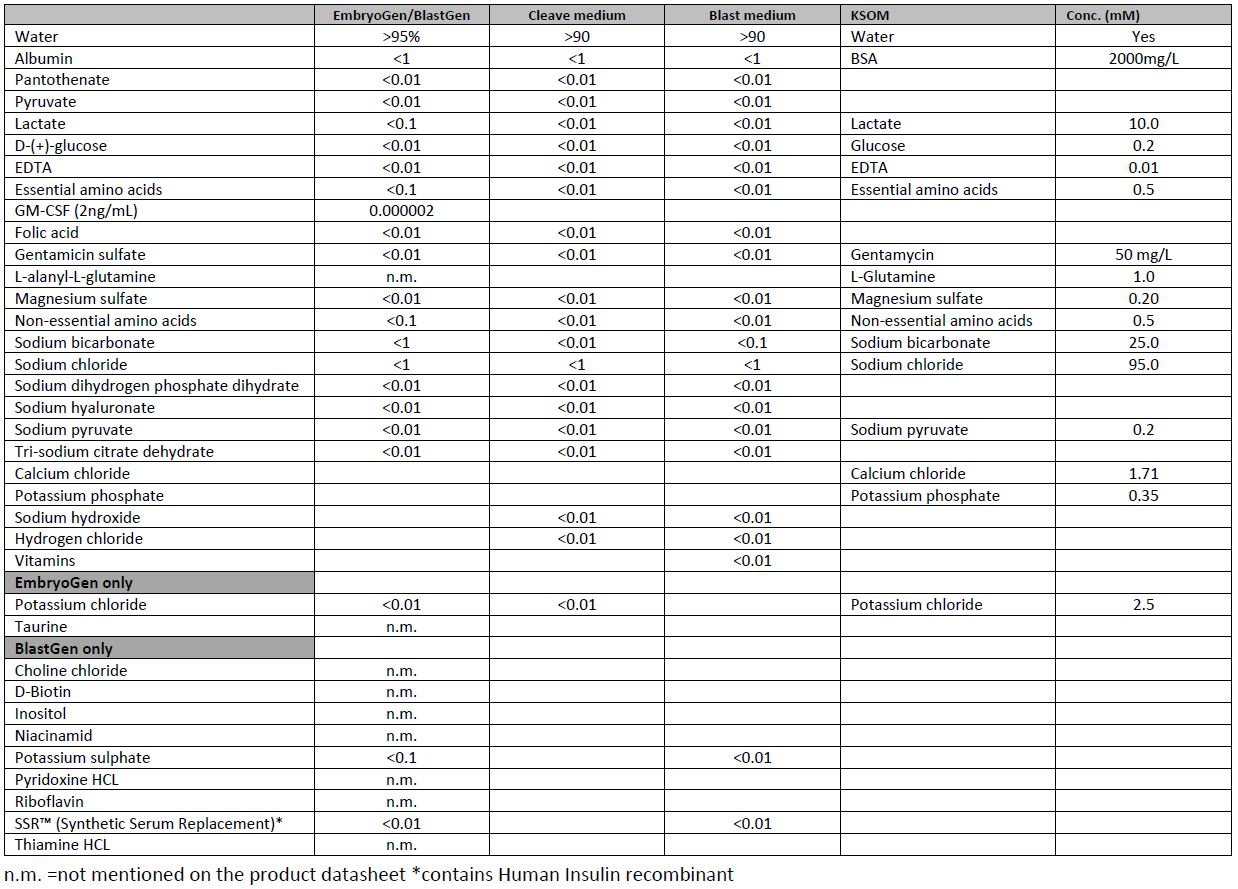

Supplement: S1 Table — Medium composition of the commercially available ART media and the control medium KSOM(aa). (TIF) [file pone.0263793.s004.tif]

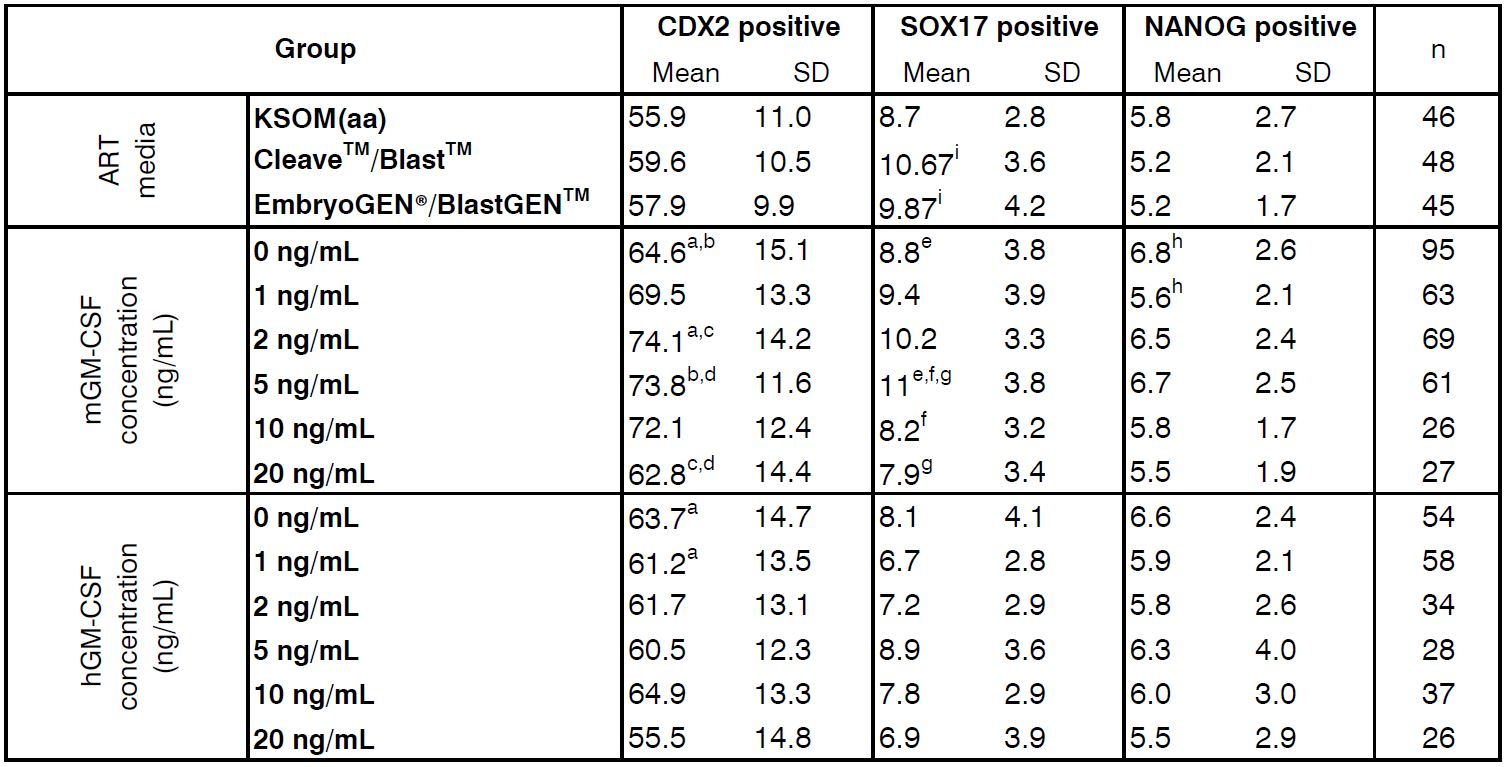

Supplement: S2 Table — Mean numbers, standard deviation and n-values of all media groups and cell lineages identified by specific cell lineage markers (CDX2: trophectoderm; SOX17: primitive endoderm; NANOG: epiblast). Significance values: a: P<0.0001; b, c, e, f: P<0.001; d, g, h, i: P<0.05; One-Way ANOVA followed by Bonferroni post-hoc test. (TIF) [file pone.0263793.s005.tif]

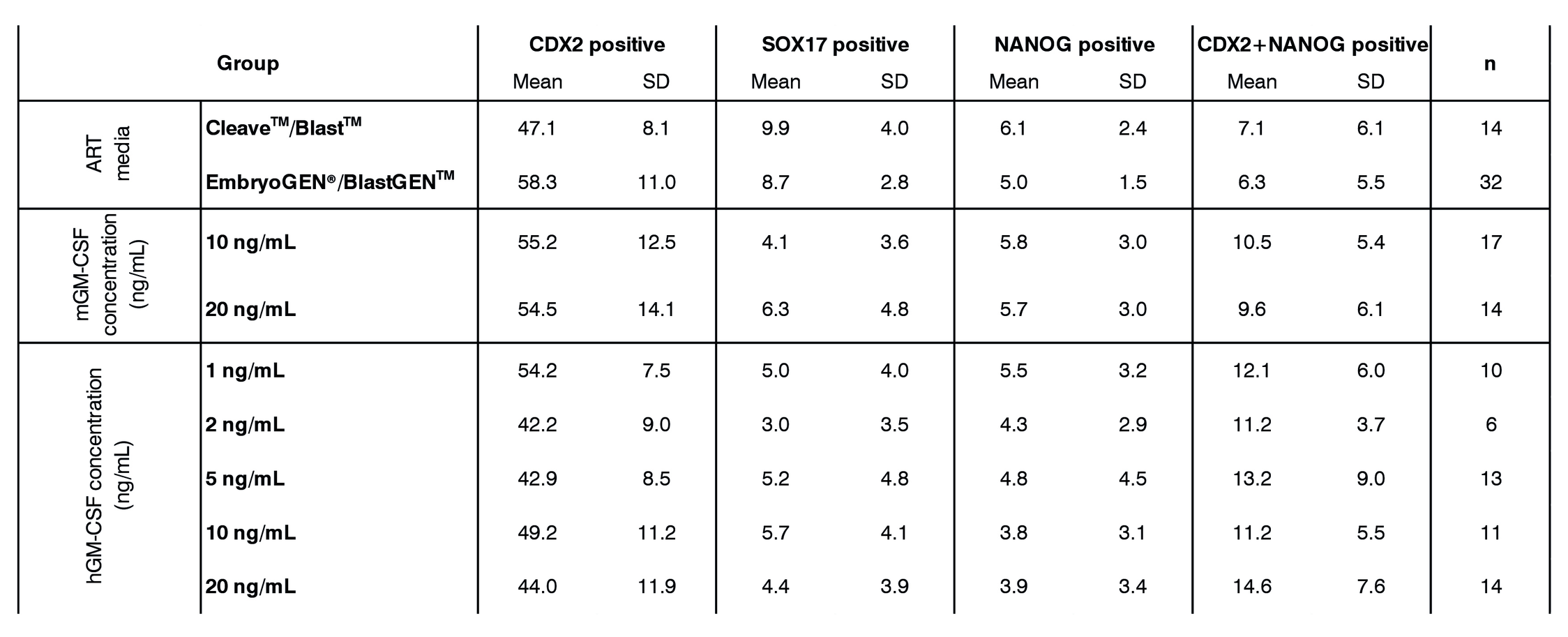

Supplement: S3 Table — Mean numbers, standard deviation and n-values of all media groups of embryos with double staining of NANOG in CDX2 positive cells, identified by specific cell lineage markers (CDX2: trophectoderm; SOX17: primitive endoderm; NANOG: epiblast). (TIF) [file pone.0263793.s006.tif]
